# Prevalence of suicidal behaviour among students living in Muslim-majority countries: systematic review and meta-analysis

**DOI:** 10.1192/bjo.2023.48

**Published:** 2023-04-14

**Authors:** S. M. Yasir Arafat, Anuradha Baminiwatta, Vikas Menon, Rakesh Singh, Natarajan Varadharajan, Saptarshi Guhathakurta, Rameez Ali Mahesar, Mohsen Rezaeian

**Affiliations:** Department of Psychiatry, Enam Medical College and Hospital, Bangladesh; Department of Psychiatry, Faculty of Medicine, University of Kelaniya, Sri Lanka; Department of Psychiatry, Jawaharlal Institute of Postgraduate Medical Education and Research, India; Department of Research, Transcultural Psychosocial Organization Nepal, Nepal; and Department of Community Medicine and Public Health, KIST Medical College, Nepal; The Banyan, Chengalpattu, India; Department of Media and Communication Studies, Shah Abdul Latif University, Pakistan; Department of Epidemiology and Biostatistics, Occupational Environmental Research Center, Rafsanjan Medical School, Rafsanjan University of Medical Sciences, Iran

**Keywords:** Suicide, Muslim countries, systematic review, meta-analysis, prevalence

## Abstract

**Background:**

Assessing suicidal behaviours among students would help to understand the burden and enhance suicide prevention.

**Aims:**

We aimed to determine the prevalence of suicidal behaviour among students living in Muslim-majority countries.

**Method:**

We followed Preferred Reporting Items for Systematic Reviews and Meta-Analyses guidelines. A systematic search was conducted in Medline, EMBASE and PsycINFO. Meta-analyses were performed to pool the lifetime, 1-year and point prevalence rates for suicidal ideation, plans and attempts.

**Results:**

From 80 studies, 98 separate samples were included in this analysis. The majority (*n* = 49) were from the Eastern Mediterranean, and 61 samples were of university students. The pooled prevalence of suicidal ideation was 21.9% (95% CI 17.4%–27.1%) for lifetime, 13.4% (95% CI 11.1%–16.1%) for the past year and 6.4% (95% CI 4.5%–9%) for current. The pooled prevalence of suicide plans was 6.4% (95% CI 3.7%–11%) for lifetime, 10.7% (95% CI 9.1%–12.4%) for the past year and 4.1% (95% CI 2.7%–6.2%) for current. The pooled prevalence of suicide attempts was 6.6% (95% CI 5.4%–8%) for lifetime and 4.9% (95% CI 3.6%–6.5%) for the past year. The lifetime prevalence of suicidal ideation was highest (46.2%) in South-East Asia, but the 12-month prevalence was highest (16.8%) in the Eastern Mediterranean.

**Conclusions:**

The study revealed notably high rates of suicidal behaviours among students living in Muslim-majority countries. However, the quality of studies, differences in regional and cultural factors, stages of studentship and methods of measurement should be considered when generalising the study results.

## Background

Suicide is the fourth leading cause of death among persons aged 15–29 years.^1^ About 77% of total suicides and 88% of suicides among adolescents occur in low- and middle-income countries (LMICs).^1^ The burden is higher when considering suicidal behaviour. Suicidal behaviour includes suicidal thoughts, plans, attempts and death by suicide.^2^ These are influenced by multifaceted interactions between biological, genetic, psychological, social and cultural factors.^3^ Research has found that the factors associated with suicidal behaviour in young people include mental health problems;^4^ alcohol and substance misuse;^5^ sexual minority status;^6,7^ familial factors such as parental loss, discord and separation;^8^ academic stress;^8,9^ economic difficulties^10^ and low social support.^11^

## Suicidal behaviour among students

Suicidal ideation not only raises the risk of suicide attempt and death from suicide,^12^ but also has associations with mental health problems, high-risk sexual practices and criminal activity.^13^ It appears to be more common among university students. According to a multi-country study, approximately 29% of university students had experienced suicidal thoughts at some point in their lives.^14^ Another multi-country survey found that 11.7% of university students had experienced suicidal thoughts at some point in their lives.^15^

There are about 50 Muslim-majority countries/territories in the world, most with LMIC backgrounds.^16–18^ There are religious prohibitions against suicide, and suicide is considered a criminal offence in several Islamic countries,^19^ and as a result, suicide has historically been understudied in these countries.^20,21^ Furthermore, many Islamic nations do not collect or submit national suicide statistics to the World Health Organization (WHO).^22^ To our knowledge, ours is the first systematic review and meta-analysis assessing the prevalence of suicidal behaviour among students in Muslim-majority countries.

## Method

### Search strategy

A systematic search was conducted in the databases Medline, EMBASE and PsycINFO, using predesigned search terms to locate articles mentioning the prevalence of suicidal behaviour among students living in Muslim-majority countries. Search details are in Supplementary File 1 available at https://doi.org/10.1192/bjo.2023.48, and we registered the protocol in advance (PROSPERO identifier CRD42022319612). The review included all of the published articles from inception to search date (10 March 2022), irrespective of the period of data collection.

### Inclusion criteria

Articles reporting results from original research studies that had cross-sectional designs, included quantitative estimates of rates of suicidal behaviour, focused on students in Muslim-majority countries, were published in the English language and for which the full text was available were included. No age or gender restrictions were applied.

### Exclusion criteria

We excluded articles with qualitative outcomes. For multiple papers from the same project, we included the most recent and/or comprehensive paper and excluded the rest. All types of review, editorial, erratum and letters without primary data were excluded.

### Study selection

Two authors (S.M.Y.A., V.M.) screened the studies independently and a third author (A.B.) commented if any ambiguous situations arose. We followed Preferred Reporting Items for Systematic Reviews and Meta-Analyses (PRISMA) guidelines and the stepwise details of the search are described in Supplementary File 2.

### Data extraction

We extracted the name of the lead authors, year of publication, country where the study was conducted, WHO region of the country, name of the journal, place of study, instruments measuring suicidal behaviour, study duration, data collection year, study design, data collection methods, study setting (rural/urban), level of studentship (elementary school/high school (college)/university), sources of the case, sample size, male/female ratio and rates of suicidal ideation, suicide plan, suicide attempts and non-suicidal self-injury (NSSI) in the lifetime, past year and during the current time (study period). We extracted data from Muslim countries in the studies conducted in multiple countries and considered a different set of data. The data yielded 80 studies from which 98 separate samples were included in this review. Among the 50 Muslim territories, studies were identified from 19 countries (Azerbaijan, Bangladesh, Brunei Darussalam, Egypt, Indonesia, Iran, Jordan, Kuwait, Libya, Lebanon, Malaysia, Morocco, Palestine, Pakistan, Sierra Leone, Saudi Arabia, Turkey, Tunisia and United Arab Emirates). Two authors (S.T. and R.A.M.) separately extracted the data in Microsoft Excel version 10 for Windows, and a third opinion from another author (S.M.Y.A.) was taken when necessary.

### Quality assessment

The methodological quality of the studies was assessed using the Newcastle–Ottawa Scale, adapted for cross-sectional studies.^23^ The tool includes three parameters: selection (representativeness of the sample, sample size, non-respondents, ascertainment of exposure), comparability (comparability of individuals in different outcome groups on the basis of design or analysis) and outcome (assessment of outcome, statistical test). Two of the authors (S.M.Y.A. and N.V.) examined the full texts of the included articles to categorise every study on these parameters. A score of ‘1’ or ‘0’ was given under each parameter depending on whether the criteria were satisfied or not, respectively, as per the manual of the Newcastle–Ottawa Scale. In some studies, when the criteria were satisfied with a validated method, a score of ‘2’ was given. The sum of scores for all subscale items was used to categorise overall study quality as either high (>7), moderate (5–7) or low (<5). Any disagreement was resolved by mutual discussion among senior authors.

### Data analysis

The prevalence estimates in the selected studies for different suicidal behaviours (ideation, plan and attempt) and NSSI were meta-analysed to create pooled prevalence estimates with the ‘Meta’ and ‘Metafor’ packages in RStudio version 1.4.1717 for Windows (Posit Software PBC, Boston, USA; http://www.rstudio.com/). The random intercept logistic regression method was used to pool the data. Heterogeneity was examined with the *I*²-statistic. Because of the high heterogeneity, random-effects models were used for the syntheses. Pooled results were displayed with forest plots. Subgroup analyses were performed to see whether the prevalence estimates varied across three settings: university, high school and elementary school. Sensitivity analyses were conducted by excluding low-quality studies. Moderator analyses were performed to test the moderating effect of gender composition (i.e. percentage of males) and the year of publication on effect sizes. Publication bias was analysed by inspecting the funnel plots, and the Egger's test was used for funnel plot asymmetry; a significant *P*-value (<0.05) for Egger's test indicated the presence of publication bias. Subgroup analyses, moderation analyses and tests of funnel plot asymmetry were not performed in meta-analyses with fewer than ten studies. Only those subgroups having at least two studies were included in the subgroup analysis.

### Ethical aspects

We reviewed secondary data from publicly available articles. Therefore, no institutional review board approval was sought to conduct the study.

## Results

### Characteristics of included studies

A total of 80 studies were included in this review. In cross-country studies, the population in each country was considered as a separate population when conducting meta-analyses; thus, a total of 98 separate samples were included in the analyses. The characteristics of these studies are summarised in Table 1. The majority of these populations were from the Eastern Mediterranean (*n* = 49), followed by Europe (*n* = 23), South-East Asia (*n* = 18), the Western Pacific (*n* = 7) and Africa (*n* = 1). With regard to individual countries, the highest number of studies was obtained from Turkey (*n* = 22), followed by Iran (*n* = 15) and Bangladesh (*n* = 11).

**Table 1:** Characteristics of studies

Serial number	Study	Region	Country	Study setting	Instruments	Study duration	Data collection year	Data collection methods	Level of study	Sources of cases	Suicidal behaviour	Number of cases	Male:female ratio	Quality appraisal
1	Abdeen et al, 2018^24^	EMR	Palestine	Urban	Health Behavior in School Aged Children in the Middle East Study Scale	5 months		Survey	School	93 single-gender and seven coeducation schools	Ideation and attempt	5713	0.44:1	Moderate
2	Afifi, 2004^25^	EMR	Egypt	Urban	Attitude Towards Suicide Scale	1 year	1996	Survey	High school	12 schools in six districts of Alexandria	Ideation and attempt	1621	1.03:1	Moderate
3	Ahmad et al, 2014^26^	WPR	Malaysia	Nationwide		2 months, 3 days	2012	Secondary analysis of national GSHS data	School	234 government schools	Ideation	25174	0.96:1	Moderate
4	Ahmadpoor et al, 2021^27^	EMR	Iran	Urban		1 year, 3 months	2017	Survey	University	13 medical universities	Ideation and attempt	4261	1.26:1	Moderate
5	Ahmed et al, 2016^28^	EMR	Egypt	Urban	BSSI	1 month	2016	Survey (online)	University	Undergraduate medical students	Ideation and plan	612	0.45:1	Moderate
6	Asante et al, 2021^29^	AFR	Sierra Leone		Single item question from GSHS questionnaire		2017	Secondary analysis of national GSHS data	School	Secondary school	Ideation and attempt	2798	0.87:1	Moderate
7	Aldalaykeh et al, 2020^30^	EMR	Jordan	Urban				Survey	University	Public university	Attempt	160	0.66:1	Low
8	Almoammar et al, 2021^31^	EMR	Saudi Arabia	Urban		3 months	2020	Survey (online)	University	Dental students and interns	Ideation and attempt	218	0.73:1	Moderate
9	Amiri et al, 2013^32^	EMR	United Arab Emirates	Urban				Survey	University	University students at the Faculty of Medicine and Health Sciences	Ideation and attempt	115	0.69:1	Moderate
10	Arafat et al, 2022^33^	SEAR	Bangladesh	Urban		3 months	2021	Survey (online)	University	Medical college and university students	Attempt	529	1.07:1	Low
11	Akpinar Aslan et al, 2020^34^	EUR	Turkey	Urban	MINI			Survey	University	Turkish university freshmen students	Attempt	355	0.37:1	Moderate
12	Atlam et al, 2017^35^	EUR	Turkey	Urban	Addiction Profile Index- short form	1 year		Survey (online)	University	Undergraduate and postgraduate university students	Attempt and plan	2973	0.82:1	Moderate
13	Azami and Taremian, 2020^36^	EMR	Iran	Urban			2019	Survey	High school	High school students	Attempt	400	0.84:1	Moderate
14	Badr, 2017^37^	EMR	Kuwait		Three items from GSHS questionnaire	12 months	2010–2011	Secondary analysis of national GSHS data	School	Adolescents aged 13–15 years	Ideation, plan and attempt	2672	1.01:1	Moderate
15	Badr and Francis, 2018^38^	EMR	Kuwait		Kuwait GSHS questionnaire	12 months		Secondary analysis of national GSHS data	School	Adolescents aged 13–15 years	Ideation, plan and attempt	1310		High
16	Bibi et al, 2021^39^	EMR	Pakistan		SBQ-R			Survey (online)	University	University students	Ideation and attempt	355	0.45:1	Low
17	Canbaz and Terzi, 2018^40^	EUR	Turkey	Urban	YRBSS	2 months	2015	Survey	High school	High school	Ideation	2438	0.97:1	Moderate
18	Chen et al, 2005^41^	WPR	Malaysia	Both	Malaysian Modified YRBSS	3 months	2001	Survey	School	Schools	Ideation, plan and attempt	4500	0.86:1	Low
19	Çimen et al, 2017^42^	EUR	Turkey	Urban	Inventory of Statements About Self-injury			Survey	School	School students of 7th–10th grades	NSSI	555	0.76:1	Low
20	Dalkilic et al, 2013^43^	EUR	Turkey	Urban	CES-D	2 months	2010	Survey	High school	High school students	Ideation	28303	0.84:1	Moderate
21	Elhadi et al, 2020^44^	EMR	Libya	Urban		10 days	2020	Survey	University	Medical students	Ideation	2430	0.26:1	Low
22	El- Matury, 2021^45^	SEAR	Indonesia	Urban	C-SSRS				University	Undergraduate students at 14 colleges	Ideation and attempt	504	0.86:1	Moderate
23	Eskin et al, 2005^46^	EUR	Turkey	Urban				Survey	University	University students	Ideation and attempt	1262	0.84:1	Low
24a	Eskin et al, 2019^47^	EUR	Azerbaijan					Survey	University	University students	Suicidal ideation and attempt	711	0.96:1	Moderate
24b	Eskin et al, 2019^47^	EMR	Egypt					Survey	University	University students	Ideation and attempt	653	0.98:1	Moderate
24c	Eskin et al, 2019^47^	SEAR	Indonesia					Survey	University	University students	Ideation and attempt	300	0.44:1	Moderate
24d	Eskin et al, 2019^47^	EMR	Iran					Survey	University	University students	Ideation and attempt	700	1.0:1.0	Moderate
24e	Eskin et al, 2019^47^	EMR	Jordan					Survey	University	University students	Ideation and attempt	700	1.0:1.0	Moderate
24f	Eskin et al, 2019^47^	EMR	Lebanon					Survey	University	University students	Ideation and attempt	706	0.95:1	Moderate
24g	Eskin et al, 2019^47^	WPR	Malaysia					Survey	University	University students	Ideation and attempt	560	1.09:1	Moderate
24h	Eskin et al, 2019^47^	EMR	Pakistan					Survey	University	University students	Ideation and attempt	700	0.86:1	Moderate
24i	Eskin et al, 2019^47^	EMR	Palestine					Survey	University	University students	Ideation and attempt	793	0.66:1	Moderate
24j	Eskin et al, 2019^47^	EMR	Saudi Arabia					Survey	University	University students	Ideation and attempt	1137	0.6:1	Moderate
24k	Eskin et al, 2019^47^	EMR	Tunisia					Survey	University	University students	Ideation and attempt	707	0.99:1	Moderate
24l	Eskin et al, 2019^47^	EUR	Turkey					Survey	University	University students	Ideation and attempt	750	0.73:1	Moderate
25	Eskin et al, 2011^48^	EUR	Turkey	Urban				Survey	University	Medical students	Ideation and attempt	326	1.41:1	Low
26	Eskin et al, 2014^49^	EUR	Turkey	Urban				Survey	High school	High school students	Ideation and attempt	541	0.55:1	Low
27a	Eskin et al, 2016^14^	EMR	Iran					Survey	University	University students	Ideation and attempt	1000	0.65:1	Moderate
27b	Eskin et al, 2016^14^	EMR	Jordan					Survey	University	University students	Ideation and attempt	436	0.68:1	Moderate
27c	Eskin et al, 2016^14^	EMR	Palestine					Survey	University	University students	Ideation and attempt	358	0.67:1	Moderate
27d	Eskin et al, 2016^14^	EMR	Saudi Arabia					Survey	University	University students	Ideation and attempt	413	2.33:1	Moderate
27e	Eskin et al, 2016^14^	EMR	Tunisia					Survey	University	University students	Ideation and attempt	484	0.29:1	Moderate
27f	Eskin et al, 2016^14^	EUR	Turkey					Survey	University	University students	Suicidal ideation and attempt	497	0.59:1	Moderate
28	Eskin et al, 2007^50^	EUR	Turkey	Urban				Survey	High school	High schools	Ideation and attempt	805	1.19:1	Moderate
29	Evren et al, 2014^51^	EUR	Turkey	Urban	Adapted from ESPAD survey questionnaire	3 months	2012	Survey (online)	School	Schools	Ideation and attempt	4957	0.82:1	High
30	Fekih-Romdhane et al, 2020^52^	EMR	Tunisia	Urban	Suicidal Ideation Questionnaire			Survey	University	Medical students	Ideation and attempt	390	0.34:1	Moderate
31	Ghahremani et al, 2019^53^	EMR	Iran	Urban	Modified adolescents high-risk behavior questionnaire				High school	High school students	Ideation, plan and attempt	483	1.65:1	Moderate
32	Ghazanfar et al, 2015^54^	EMR	Pakistan	Urban		31 months	2012–2014	Survey	University	Medical students	Ideation	1132	0.92:1	Low
33	Gholamrezaei et al, 2016^55^	EMR	Iran	Urban	SBQ-R and NSSI Scale			Survey	University	University students	NSSI and attempt	554	0.75:1	Moderate
34	Ghrayeb et al, 2014^56^	EMR	Palestine	Urban	Arabic version of GSHS			Secondary analysis of national GSHS data	High school	High school students	Ideation and attempt	720	1.01:1	Low
35	Guedria-Tekari et al, 2019^57^	EMR	Tunisia	Urban	SBQ-R	2 months	2012	Survey	High school	High school students	Ideation and attempt	821	0.47:1	Moderate
36	Hamdan and Hallaq, 2021^58^	EMR	Palestine	Urban	C-SSRS, and SBQ-R			Survey	University	University and college students	Ideation and attempt	303	0.94:1	Moderate
37	Hasan et al, 2022^59^	SEAR	Bangladesh	Urban		6 months	2013		University	Undergraduate medical students	Ideation and attempt	221	0.63:1	Moderate
38	Ibrahim and Mahfoud, 2021^60^	EMR	United Arab Emirates	Urban				Secondary analysis of national GSHS data	School	School students between grades 7 and 12	Ideation, plan and attempt	5826	0.91:1	High
39	Idig-Camuroglu and Gölge, 2018^61^	EUR	Turkey	Urban	Inventory of Statements about Self-Injury			Survey	University	University students	NSSI	1000	0.45:1	Moderate
40	Irish and Murshid, 2020^62^	SEAR	Bangladesh		GSHS survey questionnaire			Secondary analysis of national GSHS data	School	School students of classes 7 to 10	Attempt	2883	0.67:1	Moderate
41	Itani et al, 2017^63^	EMR	Palestine		GSHS survey questionnaire	Not applicable	2010	Secondary analysis of national GSHS data	School	Students aged 13– 15 years, studying in grades 7–9 in schools	Ideation	14,303	0.89:1	Moderate
42	Itani et al, 2018^64^	EMR	United Arab Emirates		GSHS survey questionnaire			Secondary analysis of national GSHS data	School	Adolescent school students aged 13–17 years	Ideation	2520	0.71:1	Moderate
43	Izadi-Mazidi et al, 2019^65^	EMR	Iran	Urban	Persian version of Functional Assessment of Self-Mutilation	5 months	2018	Survey	School	School students aged 15–18 years	NSSI	646	1.19:1	Moderate
44	Karbeyaz et al, 2016^66^	EUR	Turkey	Urban		12 years		Nationalised record data	University	Deceased students	Suicide	75	1.2:1	Low
45	Khan et al, 2020^67^	SEAR	Bangladesh		GSHS survey questionnaire			Secondary analysis of national GSHS data	School	School students aged 11–17 years	Ideation, plan and attempt	2989	1.88:1	High
46	Khokher and Khan, 2005^68^	EMR	Pakistan		GHQ-28			Survey	University	Medical students	Ideation	217	0.79:1	Low
47	Khosravi and Kasaeiyan, 2020^69^	EMR	Iran	Urban	BSSI	3 months		Survey	University	Medical students	Ideation	376	1	High
48	Klibert et al, 2021^70^	EMR	Pakistan	Urban	SBQ-R			Survey	University	University students	Ideation, plan and attempt	449	0.18:1	Moderate
49	Madadin et al, 2021^71^	EMR	Saudi Arabia	Urban	GHQ-28			Survey (online)	University	Medical students	Ideation	265	0.67:1	Low
50	Mamun et al, 2022^72^	SEAR	Bangladesh	Urban		2 months	2019	Survey	University	University students	Ideation	665	2.1:1	Moderate
51	Mamun et al, 2022^73^	SEAR	Bangladesh	Urban		10 days	2019	Survey	University	University students	Ideation, plan and attempt	911	1.16:1	Moderate
52	Marin et al, 2020^74^	EMR	Iran	Urban			2017–2018	Survey	High school	High school students (aged 14–18 years)	NSSI	6229	0.88:1	Moderate
53	Mousavi et al, 2012^75^	EMR	Iran	Urban	BSSI	8 months		Survey	University	University students	Ideation	435	0.71:1	Moderate
54	Nemati et al, 2020^76^	EMR	Iran	Urban			2017	Survey	High school	High school students	NSSI	3966	0.89:1	Low
55	Osama et al, 2014^77^	EMR	Pakistan	Urban		1 month	2013	Survey	University	Medical students	Ideation, plan and attempt	331	0.7:1	Moderate
56	Oksuz and Malhan, 2005^78^	EUR	Turkey	Urban	YRBSS	8 months	2003	Survey	University	University students	NSSI and attempt	640	0.96:1	Moderate
57	Oyekcin et al, 2017^79^	EUR	Turkey	Urban		9 months	2011–12	Survey (online)	University	University students	Ideation	4428	0.87:1	Low
58	Payci et al, 2005^80^	EUR	Turkey	Urban	Turkish version of YRBSQ		1999–2000	Survey	High school	High school students	Ideation, plan and attempt	2352	1	Moderate
59a	Peltzer and Pengpid, 2017^81^	SEAR	Indonesia		GSHS survey questionnaire		2007	Secondary analysis of national GSHS data	School	School	Ideation	2867	0.98:1	Moderate
59b	Peltzer and Pengpid, 2017^81^	WPR	Malaysia		GSHS survey questionnaire		2012	Secondary analysis of national GSHS data	School	School	Ideation	16095	0.98:1	Moderate
60a	Peltzer and Pengpid, 2017^15^	SEAR	Indonesia	Urban			2015	Survey	University	University students	Ideation and attempt	231	0.31:1	Moderate
60b	Peltzer and Pengpid, 2017^15^	WPR	Malaysia	Urban			2015	Survey	University	University students	Ideation and attempt	1023	0.97:1	Moderate
61	Pengpid and Peltzer, 2020^82^	SEAR	Indonesia		GSHS		2015	Secondary analysis of national GSHS data	School	School	Ideation, plan and attempt	11105		Moderate
62	Poorolajal et al, 2019^83^	EMR	Iran	Urban		15 months	2016–2017	Survey	University	Medical students	Ideation and attempt	4261	0.79:1	Moderate
63	Putra et al, 2019^84^	SEAR	Indonesia		GSHS questionnaire		2015	Secondary analysis of national GSHS data	School	School students	Ideation and attempt	8634	0.73:1	Moderate
64	Rahman et al, 2022^85^	SEAR	Bangladesh	Urban		5 months	2019	Survey	University	University students	Ideation	407	1.18:1	Moderate
65	Rahman et al, 2022^86^	SEAR	Bangladesh	Urban	SBQ-R	16 days	2021	Survey (online)	University	University students	Suicidal behaviour	2100	1.27:1	Moderate
66	Rasheduzzaman et al, 2022^87^	SEAR	Bangladesh	Urban		2 months	2019	Survey	University	University students	Ideation, plan and attempt	1844	2.25:1	Moderate
67	Sadeghian et al, 2021^88^	EMR	Iran	Urban		12 months	2018	Survey	University	Medical students	Ideation and attempt	224	0.6:1	Moderate
68	Sakib et al, 2021^89^	SEAR	Bangladesh	Urban	Patient Health Questionnaire (PHQ-9)	3 months	2019	Survey	University	University Students	Suicidal behaviour	955	1.04:1	Low
69	Shahedifar et al, 2020^90^	WPR	Brunei Darussalam		GSHS		2014	Secondary analysis of national GSHS data	School	School students	Ideation, plan and attempt	2599	1	Moderate
70	Somer et al, 2015^91^	EUR	Turkey	Urban	Inventory of Statements About Self-Injury, Suicide Probability Scale		2010–2011	Survey	High school	High school students	NSSI	1656	0.82:1	Moderate
71	Tan et al, 2015^92^	WPR	Malaysia	Urban	SBQ-R	2 months	2013	Survey	University	Medical students	Suicidal behaviour	537	0.54:1	Moderate
72	Tasnim et al, 2020^93^	SEAR	Bangladesh			2 months	2020	Survey (online)	University	University students	Ideation and attempt	3331	1.5:1	Moderate
73	Toprak et al, 2011^94^	EUR	Turkey	Urban		13 months	2007–08	Survey	University	University students	NSSI, ideation and attempt	636	0.85:1	High
74	Toros et al, 2004^95^	EUR	Turkey	Both				Survey	School	School students	Attempt	4143		High
75	Tresno et al, 2012^96^	SEAR	Indonesia	Urban	The Deliberative Self-Harm Inventory			Survey	University	University students	NSSI and attempt	307	0.31:1	Low
76	Vasegh and Ardestani, 2018^97^	EMR	Iran					Survey	University	University students	Ideation, plan and attempt	421	0.48:1	Moderate
77	Vehid et al, 2006^98^	EUR	Turkey	Urban	BDI			Survey	School	School students	Ideation	3609	1.11:1	Moderate
78	Zarrouq et al, 2015^99^	EMR	Morocco	Urban	MINI (Moroccan Arabic version)	8 months	2012–13	Survey	High school	High school Students	Ideation, plan and attempt	3020	1.13:1	Low
79	Ziaei et al, 2017^100^	EMR	Iran		GSHS questionnaires		2013–2014	Secondary analysis of national GSHS data	High school	High school students	Ideation	1517	.92:1	High
80	Zoroglu et al, 2003^101^	EUR	Turkey	Urban				Survey	High school	High school students	NSSI and attempt	839	0.63:1	Moderate

EMR, Eastern Mediterranean region; WPR, Western Pacific region; GSHA, Global School-Based Student Health Survey; BSSI, Beck Scale for Suicide Ideation; AFR, African region; SEAR, South-East Asian region; MINI, Mini International Neuropsychiatric Interview; EUR, European region; SBQ-R, Suicidal Behaviors Questionnaire-Revised; YRBSS, Youth Risk Behavior Surveillance System; NSSI, non-suicidal self-injury; CES-D, XXX; C-SSRS, Columbia Suicide Severity Rating Scale; ESPAD, European School Survey Project on Alcohol and Other Drugs; GHQ-28, General Health Questionnaire 28; YRBSQ, Youth Risk Behavior Survey Questionnaire; BDI, Beck Depression Inventory.

A wide range of instruments had been used in the included studies for the assessment of suicidal behaviours and/or NSSI. The most frequently used instrument was the Global School-Based Student Health Survey (GSHS), which was used in 14 studies. The next most commonly used scale was the Suicidal Behaviors Questionnaire-Revised (SBQ-R), used in seven studies.

The majority of samples were composed of university students (*n* = 61); 15 were composed of high school students and 22 were composed of elementary school students. The size of individual study samples ranged from 75 to 28 303. The percentage of males in the samples ranged from 15.3% to 100%, with a median of 46.3%.

### Pooled prevalence rates of suicidal behaviours

#### Prevalence of suicidal ideation

**Fig. 1:**
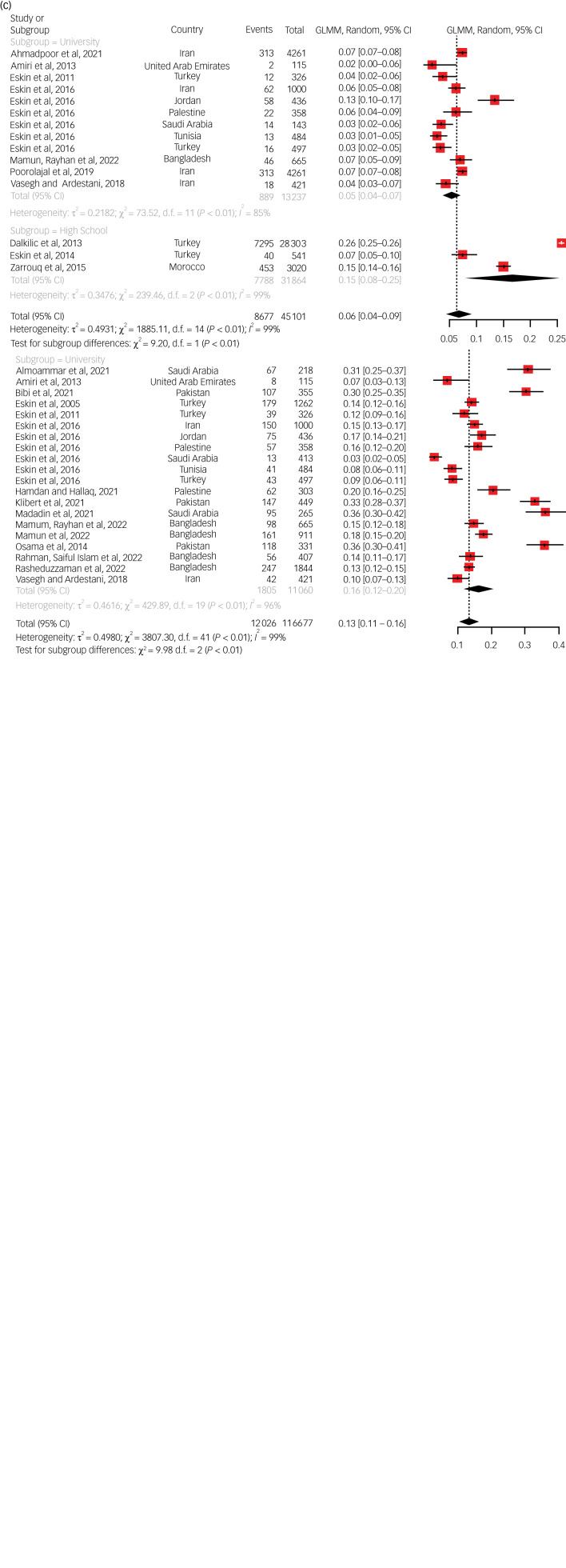
Pooled estimate for the prevalence of suicidal ideation in the lifetime, past year and current time. (a) Pooled estimate for the prevalence of suicidal ideation in the lifetime. (b). Pooled estimate for the prevalence of suicidal ideation in the past year. (c) Pooled estimate for the prevalence of suicidal ideation in the current time. GLMM, generalised linear mixed model.

The pooled lifetime prevalence of suicidal ideation among students overall was 21.9% (95% CI 17.4%–27.1%). The majority of studies (31) reporting a lifetime prevalence of suicidal ideation was conducted among university students, but a few (3) were conducted among high school students (Fig. 1). The prevalence in these two subgroups was similar. No corresponding studies among elementary school students were available. The 12-month prevalence of suicidal ideation among students overall was 13.4% (95% CI 11.1%–16.1%). High school students exhibited the highest prevalence (16.6%) out of the three subgroups, whereas elementary school students had the lowest (9.3%). This subgroup difference was statistically significant (*P* = 0.0068) (Table 2). The point prevalence of suicidal ideation overall was 6.4% (95% CI 4.5%–9%), and it was higher among high school students (14.7%) compared with university students (5.2%). This subgroup difference was statistically significant (*P* = 0.0024). No studies among elementary school students were available in this regard.

**Table 2:** Meta-analysis summary

Suicidal behaviour	Time period	Pooled prevalence [95% CI], number of studies (*k*), total population (*n*) and heterogeneity (*I*^2^)				Test of subgroup differences	Tests of moderation		Egger's test of funnel plot asymmetry
		Overall	University	High school	School		Male percentage	Year of publication	
Suicidal ideation	Lifetime prevalence	21.9% [17.4%– 27.1%], *k =* 32, *n* = 23075, *I*^2^ = 97.9%	21.3% [16.6%–27.1%], *k =* 29, *n* = 20908, *I*^2^ = 98%	26.9% [22.9%–31.2%], *k =* 3, *n* = 2167, *I*^2^= 85.7%	Not applicable	Q = 2.45 *P* = 0.1178	QM = 1.85, *P* = 0.1731	QM = 1.1, *P* = 0.2940	*t* = –1.89, *P* = 0.0681
	12-month prevalence	13.4% [11.1%–16.1%], *k =* 42, *n* = 11 6677, *I*^2^ = 98.9%	15.9% [12.2%–20.4%], *k =* 20, *n* = 94 802, *I*^2^ = 95.6%	16.6% [11.1%–24.2%], *k* = 8, *n* = 10 815, *I*^2^ = 98%	9.3% [7.1%–12.1%], *k =* 14, *n* = 11 060, *I*^2^ = 99.1%	Q = 9.98, *P* = 0.0068	QM = 1.89, *P* = 0.1687	QM = 0.077, *P* = 0.781	*t* = 2.19, *P* = 0.0348
	Point prevalence	6.4% [4.5%–9%], *k =* 15, *n* = 45 101, *I*^2^ = 99.3%	5.2% [3.9%–6.9%], *k =* 12, *n* = 13 237, *I*^2^ = 85%	14.7% [8.1%–25.3%], *k* = 3, *n* = 31 864, *I*^2^ = 99.2%	Not applicable	Q = 9.20, *P* = 0.0024	QM = 0.22, *P* = 0.6391	QM = 0.0029, *P* = 0.9573	*t* = –5.10, *P* = 0.0002
Suicide plans	Lifetime prevalence	6.4% [3.7%–11%], *k =* 3, *n* = 2877, *I*^2^ = 92.9%	6.4% [3.7%–11%], *k =* 3, *n* = 2877, *I*^2^ = 92.9%	Not applicable	Not applicable	Not applicable	Not applicable	Not applicable	Not applicable
	12-month prevalence	10.7% [9.1%–12.4%], *k =* 9, *n* = 21 312, *I*^2^ = 92.1%	9.6% [7.1%–12.7%], *k =* 3, *n* = 1663, *I*^2^ = 81.2%	14.1% [12.8%–15.6%], *k =* 1, *n* = 2352, *I*^2^ = Not applicable	10.6% [8.9%–12.7%], *k =* 5, *n* = 17 297, *I*^2^ = 93.9%	Not applicable	Not applicable	Not applicable	Not applicable
	Point prevalence	4.1% [2.7%–6.2%], *k =* 3, *n* = 4053, *I*^2^ = 84.6%	3.1% [2.2%–4.4%], *k =* 2, *n* = 1033, *I*^2^ = 0%	6% [5.2%–6.9%], *k =* 1, *n* = 3020, *I*^2^ = Not applicable	Not applicable	Not applicable	Not applicable	Not applicable	Not applicable
Suicidal attempts	Lifetime prevalence	6.6% [5.4%–8%], *k =* 34, *n* = 26 307, *I*^2^ = 93.2%	6.6% [5.4%–8.1%], *k =* 31, *n* = 21 941, *I*^2^ = 93%	6.8% [3%–14.6%], *k =* 3, *n* = 4366, *I*^2^ = 95.5%	Not applicable	Q = 0, *P* = 0.9606	QM = 1.8563, *P* = 0.1731	QM = 1.101, *P* = 0.294	*t* = –1.89, *P* = 0.0681
	12-month prevalence	4.9% [3.6%–6.5%], *k =* 32, *n* = 77 404, *I*^2^= 98.9%	3% [2.1%–4.3%], *k =* 15, *n* = 15 379, *I*^2^ = 94.5%	6.4% [2.7%–14.5%], *k =* 5, *n* = 6039, *I*^2^= 98.6%	7.5% [5.2%–10.8%], *k =* 12, *n* = 55 986, *I*^2^ = 99.4%	Q = 12.8 *P* = 0.0017	QM = 0.8621, *P* = 0.3532	QM = 0.6957, *P* = 0.4042	*t* = −2.66, *P* = 0.0124
Non-suicidal self-injury	Lifetime prevalence	16.5% [9.5%–27.2%], *k =* 6, *n* = 13 713, *I*^2^= 99.5%	28.8% [26.5%–31.4%], *k =* 2, *n* = 1307, *I*^2^ = 0%	12.4% [5.2%–26.8%], *k =* 3, *n* = 11 851, *I*^2^ = 99.7%	11.4% [9%–14.3%], *k =* 1, *n* = 555, *I*^2^ = Not applicable	Not applicable	Not applicable	Not applicable	Not applicable
	12-month prevalence	10.2% [2.1%–37.7%], *k =* 2, *n* = 6875, *I*^2^= 99.8%	Not applicable	3.3% [2.9%–3.8%], *k =* 1, *n* = 6229, *I*^2^ = Not applicable	27.5% [24.3%–31.1%], *k =* 1, *n* = 646, *I*^2^ = Not applicable	Not applicable	Not applicable	Not applicable	Not applicable

#### Prevalence of suicide plans

**Fig. 2:**
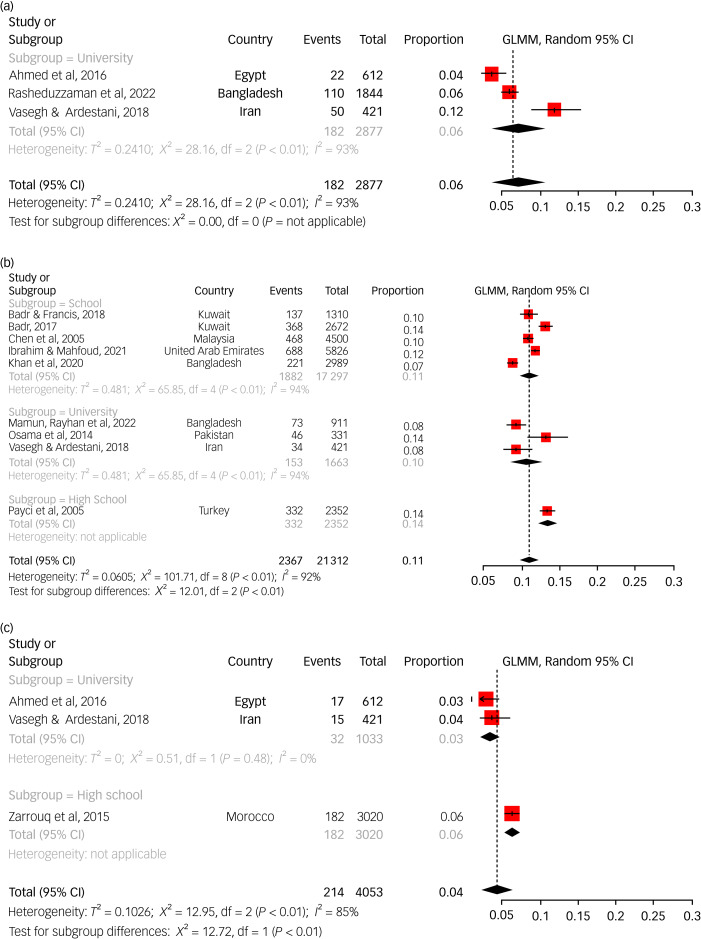
Pooled estimate for the prevalence of suicide planning in lifetime, past year and current time. (a) Pooled estimate for the prevalence of suicide planning in the lifetime. (b) Pooled estimate for the prevalence of suicide planning in the past year. (c) Pooled estimate for the prevalence of suicide planning in the current time. GLMM, generalised linear mixed model.

The lifetime prevalence of suicide plans was reported in three studies, and the pooled prevalence rate was 6.4% (95% CI 3.7%–11%). All of these three studies were conducted among university students. Nine studies reported the 12-month prevalence of suicide plans, and their pooled prevalence rate was 10.7% (95% CI 9.1%–12.4%). Out of the three subgroups, the highest prevalence was observed among high school students (Fig. 2). A point prevalence of suicide plans was reported by three studies, generating a pooled prevalence of 4.1% (95% CI 2.7%–6.2%). No studies reporting a point prevalence of suicide plans among elementary school students were found.

#### Prevalence of suicide attempts

**Fig. 3:**
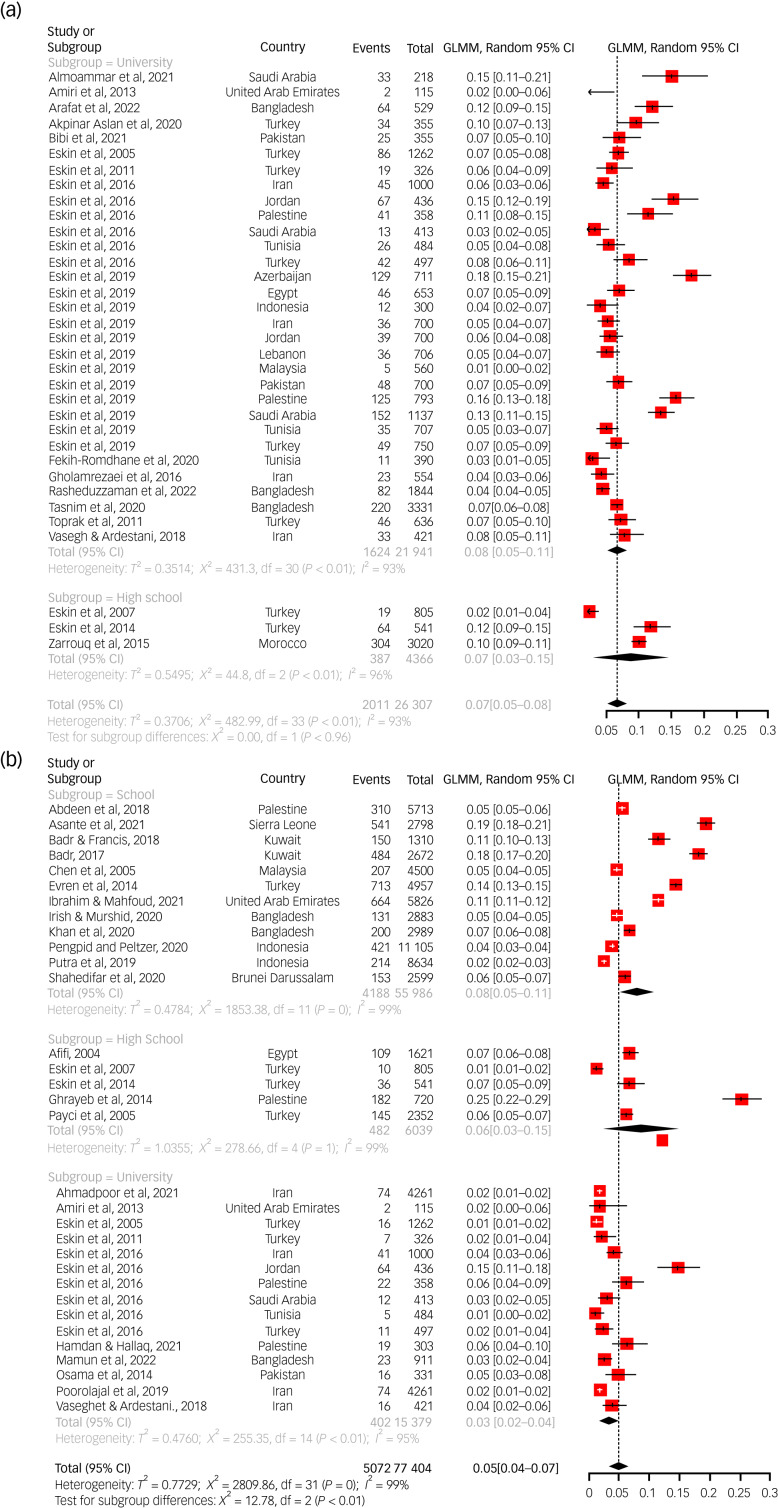
Pooled estimate for the prevalence of suicidal attempt in the lifetime and past year. (a) Pooled estimate for the prevalence of suicidal attempt in the lifetime. (b) Pooled estimate for the prevalence of suicidal attempt in the past year. GLMM, generalised linear mixed model.

The lifetime prevalence of suicide attempts was reported in 34 studies, and their pooled prevalence rate was 6.6% (95% CI 5.4%–8%). The majority of these studies (31) were conducted among university students, with three studies being conducted on high school students. There was no statistically significant difference between these two subgroups. No studies in this regard were available among elementary school students. Thirty-two studies reported the 12-month prevalence of suicidal attempts, and the pooled prevalence rate was 4.9% (95% CI 3.6%–6.5%) (Fig. 3). Statistically significant subgroup differences were observed (*P* = 0.0017); the highest prevalence was seen among elementary school students (7.5%) and the lowest rate (3%) was seen among university students.

#### Prevalence of NSSI

Six studies reported the lifetime prevalence of NSSI, and the pooled prevalence rate was 16.5% (95% CI 9.5%–27.2%). Notable subgroup differences were observed: 28.5% of university students reported a history of NSSI, and the corresponding rates among high school students and elementary school students were 12.4% and 11.4%, respectively. The 12-month prevalence of NSSI was reported by only two studies, and their pooled prevalence was 10.2% (95% CI 2.1%–37.7%) (Supplementary File 3). The prevalence rates reported by these two studies were remarkably different (27.5% *v*. 3.3%).

### Gender differences in prevalence of suicide attempts

A meta-analysis of odds ratios was conducted to calculate the pooled male/female ratio of suicide attempts among students. When suicide attempts were considered irrespective of the timeframe, the male/female ratio was 0.77 (95% CI 0.59–1.01), based on data from 19 studies. When suicide attempts during the lifetime were considered, the male/female ratio was 0.77 (95% CI 0.53–1.11), based on data from eight studies. With regard to suicide attempts during the previous 12 months, the male/female ratio was 0.83 (95% CI 0.46–1.50), based on five studies. None of these gender differences reached statistical significance.

### Regional differences

#### Suicidal ideation

Both the lifetime (*P* = 0.0432) and 12-month (*P* < 0.0001) prevalence of suicidal ideation showed significant regional differences, but the point prevalence of suicidal ideation did not (*P* = 0.8304). The lifetime prevalence was highest (46.2%) in South-East Asia, but the 12-month prevalence was highest (16.8%) in the Eastern Mediterranean (Table 3).

**Table 3:** Regional variations of prevalence of suicidal behaviour among students living in Muslim countries

Suicidal behaviour	Time period	Eastern Mediterranean	Europe	South-East Asia	Western Pacific	Africa	Test of subgroup differences^a^
Suicidal ideation	Lifetime prevalence	21.6% [17.4%–26.5%], *k =* 20	23.9% [18.8%–29.8%], *k =* 9	46.2% [26.9%–66.7%], *k =* 2	Not applicable	Not applicable	Q = 6.3; *P* = 0.0432
	12-month prevalence	16.8% [12.9%–21.6%], *k =* 22	14.6% [11.4%–18.5%], *k =* 7	8.6% [5.8%–12.6%], *k =* 8	7.9% [7.7%–8.1%], *k =* 4	14.1 [12.8%–15.4%], *k =* 1	Q = 50.5; *P* < 0.0001
	Point prevalence	6% [4.1%–8.7%], *k =* 10	7.2% [3%–16.7%], *k =* 4	6.9% [5.2%–9.1%], *k =* 1	Not applicable	Not applicable	Q = 0.37 *P* = 0.8304
Suicide plans	Lifetime prevalence	6.6% [2.8%–14.9%], *k =* 2	Not applicable	6% [5%–7.1%], *k =* 1	Not applicable	Not applicable	Not applicable
	12-month prevalence	11.7% [10.1%–13.4%], *k =* 5	14.1% [12.8%–15.6%], *k =* 1	7.5% [6.8%–8.4%], *k =* 2	10.4% [9.5%–11.3%], *k =* 1	Not applicable	Not applicable
	Point prevalence	4.1% [2.7%–6.2%], *k =* 3	Not applicable	Not applicable	Not applicable	Not applicable	Not applicable
Suicidal attempts	Lifetime prevalence	6.8% [5.4%–8.5%], *k =* 20	7.7% [5.4%–10.7%], *k =* 9	6.3% [4.1%–9.6%], *k =* 4	0.9% [0.4%–2.1%], *k =* 1	Not applicable	Q = 0.55; *P* = 0.7578
	12-month prevalence	5.5% [3.5%–8.6%], *k =* 16	3.4% [1.7%–6.7%], *k =* 7	3.9% [2.9%–5.2%], *k =* 6	5.2% [4.3%–6.1%], *k =* 2	19.3% [17.9%–20.8%], *k =* 1	Q = 3.97; *P* = 0.2644
Non-suicidal self-injury	Lifetime prevalence	7.3% [5.9%–9%], *k =* 2	22.4% [13.2%–35.3%], *k =* 3	30% [25.1%–35.3%], *k =* 1	Not applicable	Not applicable	Not applicable
	12-month prevalence	10.2% [2.1%–37.7%], *k =* 2	Not applicable	Not applicable	Not applicable	Not applicable	Not applicable

a Regions with only one study (k = 1) were omitted from the subgroup analysis.

#### Suicide plans

The number of studies reporting the prevalence of suicide plans was too few to conduct and meaningfully interpret subgroup analyses. The 12-month prevalence showed some regional variation, with the highest rate (14.1%) observed in Europe (Table 3).

#### Suicide attempts

The regional variations in the lifetime and 12-month prevalence rates of suicide attempts were not statistically significant. The highest lifetime prevalence was observed in Europe (7.7%), and the highest 12-month prevalence was seen in Africa (19.3%; finding based on a single study).

#### NSSI

The number of studies reporting the prevalence of NSSI was too few to conduct subgroup analyses and meaningfully interpret their findings. The highest lifetime prevalence of NSSI (30%) was seen in South-East Asia. Data on 12-month prevalence were available only from the Eastern Mediterranean.

### Moderator analyses

Moderator analyses were performed to test whether the gender composition (percentage of males) of study samples and the year of publication had moderating effects on prevalence estimates in the meta-analyses. The findings of these moderator analyses are included in Table 2. Moderator analysis was not done if the total number of studies in a particular meta-analysis was fewer than ten, as was seen in the meta-analyses on suicide plans and NSSI. Neither of the selected moderator variables (i.e. gender composition nor publication year) showed significant moderation effects.

### Publication bias

Publication bias was assessed with funnel plots for meta-analyses with at least ten studies. The findings of the Egger's test of funnel plot asymmetry are included in Table 2.

### Quality assessment

Among the 80 studies, two-thirds of the studies (*n* = 53) were of moderate quality, a fourth (*n* = 19) were of low quality and the remaining 10% (*n* = 8) were of high quality (Table 1). On the comparability parameter, 47 out of 72 studies (moderate and low quality) scored at least >1, whereas 25 studies scored 0. Risk of bias was noted in selected domains in all of the included studies. With respect to outcome, all of the studies had a validated outcome assessment or self-report measure and described the statistical test employed.

### Sensitivity analyses

Sensitivity analyses showed that the exclusion of low-quality studies (based on the quality assessment) from each meta-analysis did not lead to substantial changes in the pooled prevalence estimates.

## Discussion

### Main findings of the review

We aimed to provide pooled prevalence estimates of different types of suicidal behaviour among students living in Muslim-majority countries. We found that about one in five students (high school and university) had experienced suicidal ideation at some point in their lifetime. One in eight students reported experiencing suicidal ideation in the year before assessment, whereas one in 16 reported experiencing it at the time of assessment; these prevalence rates were significantly higher among high school students compared with university students. Pooled prevalence rates of lifetime suicide plans and suicide attempts were similar; about one in 16 had experienced these phenomena. One in 20 students reported making an attempt to end their life in the year before assessment; once again, the prevalence was significantly higher among elementary school students compared with university students.

One in six students reported NSSI behaviours during their lifetime, with about one in ten reporting such phenomena in the year before the assessment. Interestingly, the prevalence of NSSI behaviours was significantly higher among university students compared with school (high and elementary) students. Overall, few studies provided data for comparison of the regional prevalence of suicidal behaviours. Prevalence of lifetime suicidal ideation was highest in South-East Asia, but the corresponding 12-month prevalence rates were highest among students in Eastern Mediterranean countries; for other suicidal behaviours, the number of studies was too few to make meaningful interpretations. These findings were, largely, robust across sensitivity analyses excluding low-quality studies.

### Implications of findings

Traditionally, as suicide is proscribed in Islam, suicide rates in Muslim countries have been thought to be low. However, the data have been inconsistent,^16^ and there has been no systematic collection of national suicide data and reporting in many Muslim nations.^102^ As pointed out by Pritchard et al in their population-based study comparing differences in patterns of suicide, undetermined and accidental deaths between Islamic and Western nations, underreporting of suicides was common, with greater underreporting noted in more orthodox Islamic nations such as Middle Eastern and North African nations compared with less orthodox countries.^103^ Interestingly, underreporting of suicide was also noted in Western nations and, given the stronger cultural taboos against suicide in Muslim nations, the authors argued that this may explain the relationship between level of religiosity and underreporting of suicide.

Given this background, our findings provide the first meta-analytic evidence for suicidal behaviour among students in Muslim countries. In fact, our pooled prevalence estimates for all subtypes of suicidal phenomena were higher than what had been reported in the general population in prior national and cross-national meta-analytic reviews.^104,105^ They were also higher than figures noted in prior national and cross-national reviews on college students, particularly for suicide attempts.^106,107^ These findings suggest the need for instituting robust suicide surveillance and data-gathering mechanisms to design effective suicide prevention programmes aimed at high school and university students in Muslim-majority countries. They also suggest the need for these issues to be high on the research and policy agenda. As our results show, authorities in Muslim nations should regard suicide as a public health issue, and not minimise its extent or severity. In many Islamic nations, suicide attempt is a punishable offence; in this context, decriminalisation of attempted suicide would be a welcome step to encourage suicide reporting, and more importantly, reduce stigma and improve help-seeking behaviour.^19^ Finally, considering suicide as an outcome of social and mental health issues in Muslim nations, best practices for assessing suicide and suicide risk in a sensitive, non-judgemental manner that neither decreases the patient's self-esteem nor challenges their religious beliefs, would enhance suicide reporting and improve help-seeking behaviour. This may enhance data-gathering efforts.

The prevalence of suicidal ideation noted was higher than what was reported in three other multi-country studies, all of whom reported prevalence rates of 12%–17%.^108–110^ The prevalence figures for suicide plan were higher than those reported by McKinnon et al (5.8%-8.3%),^110^ but lower than those reported by Uddin et al (17%).^108^ With regard to lifetime suicide attempts, our pooled estimates were lower than a large, cross-national analysis from 53 LMICs (11%).^111^ Likewise, the past year attempt rates were also lower than two prior reports, also from LMICs.^106,108^ However, as mentioned before, the figures were higher than those reported in multinational analysis not restricted to low-resource settings.^106^ These variations may reflect, in part, differences in the meaning, context and attitudes toward suicide in various cultural, religious and economic settings. The increased figures of suicidal ideation and suicide attempt noted may reflect pressures of rapid socioeconomic transition in Muslim nations with attendant intergenerational conflicts, which when coupled with academic stress, can contribute to suicidal behaviours.^112^

Interestingly, although our pooled prevalence estimates for lifetime and past year suicidal ideation and lifetime suicide plans were comparable with prior cross-national reviews on college and university students, differences in prevalence rates were more pronounced for past year suicide plans and both lifetime and past year suicide attempt.^106,113^ This raises the intriguing possibility that students in Muslim countries may have higher rates of transition from suicidal ideas or plans to a suicide attempt, compared with their counterparts in other countries. This must be systematically examined in future research, along with risk factors for such transitions, as it has obvious implications for suicide prevention. Prior research has shown that the majority of transitions from ideation to attempt occur in the first year following onset of suicidal ideation. Additional risk factors identified were younger age, female gender and presence of psychiatric morbidity.^104^ These relevant cultural factors need to be investigated in this context, to identify subgroups of students at a higher risk of suicidal behaviour transition.

Prevalence of suicidal behaviours was higher among high school students compared with university students. Indeed, the figures were higher than that found in prior analyses on high school students and adolescents.^114,115^ Prior observations from Turkey, showing a high proportion of university students contemplating suicide compared with their counterparts from Western countries, are consistent with what we have found.^116^ There is a need to explore the role of commonly implicated risk factors for suicide and suicidal behaviour in this group, such as mental health issues, academic stress and exposure to violence in the school and community.^117–119^ At the same time, there is a need to assess and strengthen protective factors that may reduce the risk of suicide. Some of the evidence-based protective factors in this group are increasing levels of parental supervision, and better parent–child and school–child connectedness.^120^ Whether enrolment in a university confers additional risk over and above that experienced by peers in Muslim countries who do not attend university may be investigated, to know whether a causal relationship exists between university attendance and suicidal behaviours.

Rates of suicidal ideation were higher in the South-East Asian and Eastern Mediterranean regions. For other suicide constructs, few studies provided data for a meaningful interpretation of regional variations. Prior analysis of age-standardised suicide rates in Muslim-majority nations has also shown considerable regional variations.^16,121^ Suicide is a complex, multidimensional behaviour anchored in unique sociocultural contexts. Existing studies identified that the suicide rate is higher in African Muslim countries than in Asia and Europe.^16,121^ Further studies are warranted to explore this variation.

From a preventive standpoint, our findings highlight the importance of understanding drivers of suicidal behaviour among students living in Muslim countries. Specifically, there is a need to understand factors that may drive progression from suicidal ideation to behaviours in this group, to inform actionable strategies for the prediction and prevention of suicidal behaviour. Existing adolescent suicide prevention programmes can be divided into two types: strategies for early recognition and referral of at-risk individuals through comprehensive, periodic, school-based screening programmes; and strategies aimed at addressing risk or protective factors in this group.^122^ Given the high burden of suicide among high school students, we also recommend incorporation of life skills training, also recommended by the WHO, and stress management materials into the school curriculum.^123^ A recent review of suicide prevention strategies among university students found that gatekeeper training of peer counsellors, combining education with skills training, is effective in improving knowledge about suicide and boosting self-efficacy in suicide prevention.^124^

### Methodological considerations

Paradoxically, the 12-month prevalence of suicide plans was higher than the lifetime prevalence. A possible reason for this may be recall bias, noted in other contexts.^125^ Specifically, participants may underreport past suicide plans or behaviours, and thus, studies assessing lifetime suicide phenomena may report lower prevalence than those assessing recent phenomena. This may be particularly relevant for suicide plans, which are not as well-defined as suicidal ideation/attempt. It might be complicated by how the terminologies for suicidal thoughts and behaviours used by the researchers were understood by the students living in countries where suicide is socially proscribed and a criminal offence.

A striking finding was that most included studies were of low to moderate quality, with most studies falling short on the domains of sample representativeness. This would influence the grade of evidence and the strength of recommendations. Different studies used different instruments to assess suicidal behaviour. Further, more than half of the included studies came from the Eastern Mediterranean region. There was also a disproportionate contribution from a few countries; only one study was available from Africa. This imbalance in the geographical distribution of studies suggests the need for expanded and high-quality research on student suicide across Muslim-majority nations.

Most of the studies used self-report questionnaires, which might be prone to recall bias and social desirability bias, both of which may lead to systematic underreporting. This issue may have particular significance when assessing issues such as suicidal behaviour and mental health, both of which are issues surrounded by stigma. As such, it is possible that participants may have modified their responses to report desirable attributes that better suit their situation. Further, the use of single-item measures or selected items from a larger measure to assess suicidal behaviours, used in some studies in the present review, is prone to misclassification error and may have influenced the reported rates of suicidal behavior.^126^

### Strengths and limitations

Suicide is under-researched in Muslim-majority countries. It is the first systematic review and meta-analysis revealing higher rates of suicidal behaviour among the students of Muslim-majority countries. These findings would encourage practicing psychiatrists to assess suicidal behaviour when treating mental health issues among students. There were many limitations to the present meta-analysis. First, the quality of the majority of studies used in this meta-analysis was low or moderate, and only 10% of the studies were of high quality. This may have biased the cross-national comparisons. Second, there was a disproportionate research output from several nations, and therefore, the results may not be generalisable to other Muslim-majority nations from which there was little to no data. Third, an overwhelming majority of included studies were conducted in specific urban settings, and thus, the results may not be generalisable to other settings and regions in the countries. Fourth, the instruments measuring the suicidal behaviour varied widely. Data from included studies were mostly based on retrospective self-report of suicidal thoughts and behaviours, which may be subject to recall bias or deliberate underreporting. The latter may be particularly relevant in a study of this nature because of the religious sanctions against suicide in Islam and its criminal status in some Muslim nations.^19^ Fifth, the age of the participants varied widely in this meta-analysis, which limits the power of subgroup analyses for specific developmental time periods. We have not assessed sociodemographic or clinical risk factors for suicide, and this affects our ability to understand the broad basis of suicidal behaviour in the studied settings. Sixth, we have not studied the rates of transition from ideation to planning to attempt, as this information was not available in studies. This is an important focus for future research because of its obvious implications for suicide prevention. Finally, many subgroup analyses lacked statistical power because there were a limited number of studies that provided the necessary data, which led to a greater probability of false negative results.^127^

The study revealed notably high rates of suicidal behaviours among students living in Muslim-majority countries. The pooled lifetime prevalence of suicidal ideation, plan and attempt among students was 21.9%, 6.4% and 6.6%, respectively. However, the quality of studies, differences in regional and cultural factors, stages of studentship and methods of measurement should be considered when generalising the study results.

## Data Availability

The data that support the findings of this study are available from the corresponding author, S.M.Y.A., upon reasonable request.

## Supplementary material

For supplementary material accompanying this paper visit http://doi.org/10.1192/bjo.2023.48. click here to view supplementary material
