# Supplementary material for: Prevalence of suicidal behaviour among students living in Muslim-majority countries: systematic review and meta-analysis
Source: BJPsych Open. 2023 Apr 14;9(3):e67. doi: 10.1192/bjo.2023.48 (PMC10134265; doi:10.1192/bjo.2023.48)
Supplement: Supplementary file 1 [file bjosup.zip › S2056472423000480sup001.docx]

Supplementary file 1: Search details

For our searches of the databases, we used the following search terms:

(student*).ab OR (student*).ti

AND

(self?harm* or suicid*).ab OR (self?harm* or suicid*).ti

AND

(Afghanistan* or Albania* or Algeria* or Azerbaijan* or Bahrain* or Bangladesh* or Bosnia* or Herzegovin* or Brunei* or Burkina* or Chad* or Comor* or Djibouti* or Egypt* or Gambia* or Guinea* or Indonesia* or Iran* or Iraq* or Jordan* or Kazakhstan* or Kuwait* or Kyrgyzstan* or Leban* or Libya* or Malaysia* or Maldiv* or Mali* or Mauritania* or Mayot* or Morocc* or Niger* or Oman* or Pakistan* or Palestin* or Qatar* or Saudi Arabia* or Senegal* or Sierra Leon* or Somalia* or Syria* or Tajikistan* or Tunisia* or Turk* or Turkmenistan* or United Arab Emirates or Uzbekistan* or Western Sahara or Yemen*).ab OR (Afghanistan* or Albania* or Algeria* or Azerbaijan* or Bahrain* or Bangladesh* or Bosnia* or Herzegovin* or Brunei* or Burkina* or Chad* or Comor* or Djibouti* or Egypt* or Gambia* or Guinea* or Indonesia* or Iran* or Iraq* or Jordan* or Kazakhstan* or Kuwait* or Kyrgyzstan* or Leban* or Libya* or Malaysia* or Maldiv* or Mali* or Mauritania* or Mayot* or Morocc* or Niger* or Oman* or Pakistan* or Palestin* or Qatar* or Saudi Arabia* or Senegal* or Sierra Leon* or Somalia* or Syria* or Tajikistan* or Tunisia* or Turk* or Turkmenistan* or United Arab Emirates or Uzbekistan* or Western Sahara or Yemen*).ti

Databases: MEDLINE, EMBASE, and PsychINFO

Date range: from inception to the search date

Search date: March 10, 2022 at 4.30 PM (Dhaka time)
