# Supplementary material for: Prevalence of suicidal behaviour among students living in Muslim-majority countries: systematic review and meta-analysis
Source: BJPsych Open. 2023 Apr 14;9(3):e67. doi: 10.1192/bjo.2023.48 (PMC10134265; doi:10.1192/bjo.2023.48)
Supplement: Supplementary file 1 [file bjosup.zip › S2056472423000480sup002.docx]

Supplementary file 2: PRISMA 2020 Flow Diagram

Records removed *before screening*:

Duplicate records removed (n = 477)

Records identified from Medline (320), Embase (463), and PsycINFO (343)

**Identification of studies via databases and registers**

## Identification

Records after duplicates removed
(n =649)

Records excluded (n = 500)

1. Other language (52)
2. Book/chapter/ thesis (6)
3. Case report (6)
4. Conference/dissertation abstract (45)
5. Editorial/erratum/letter (19)
6. Didn’t address the research question (349)
7. Qualitative study (4)
8. Review article (15)
9. Veterans (4)

## Screening

Records screened
(n = 649)

Reports sought for retrieval

(n=149)

Reports not retrieved (n= 0)

Records excluded (n = 69)

1. Full text not available (6)
2. Multiple paper from same project (2)
3. Didn’t address the research question (61)

Reports assessed for eligibility
(n = 149)

## Included

Studies included in review

(n = 80)
