# Supplementary material for: Prevalence of suicidal behaviour among students living in Muslim-majority countries: systematic review and meta-analysis
Source: BJPsych Open. 2023 Apr 14;9(3):e67. doi: 10.1192/bjo.2023.48 (PMC10134265; doi:10.1192/bjo.2023.48)
Supplement: Supplementary file 1 [file bjosup.zip › S2056472423000480sup003.docx]

Supplementary file 3: Pooled estimate for the prevalence of NSSI


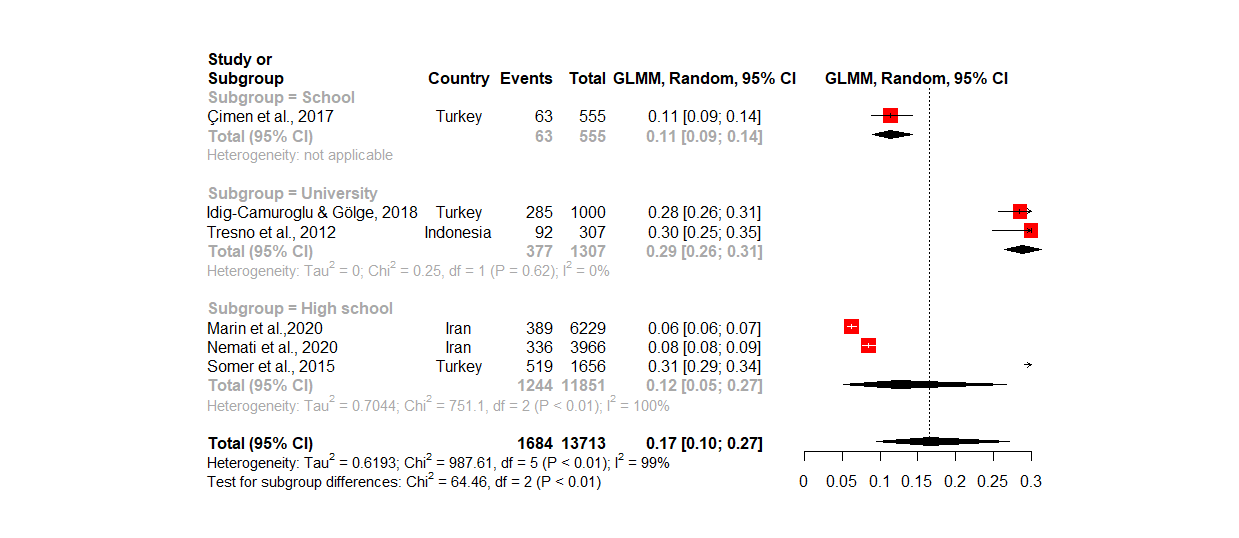


Supplementary file 3A: Pooled estimate for the prevalence of NSSI in lifetime


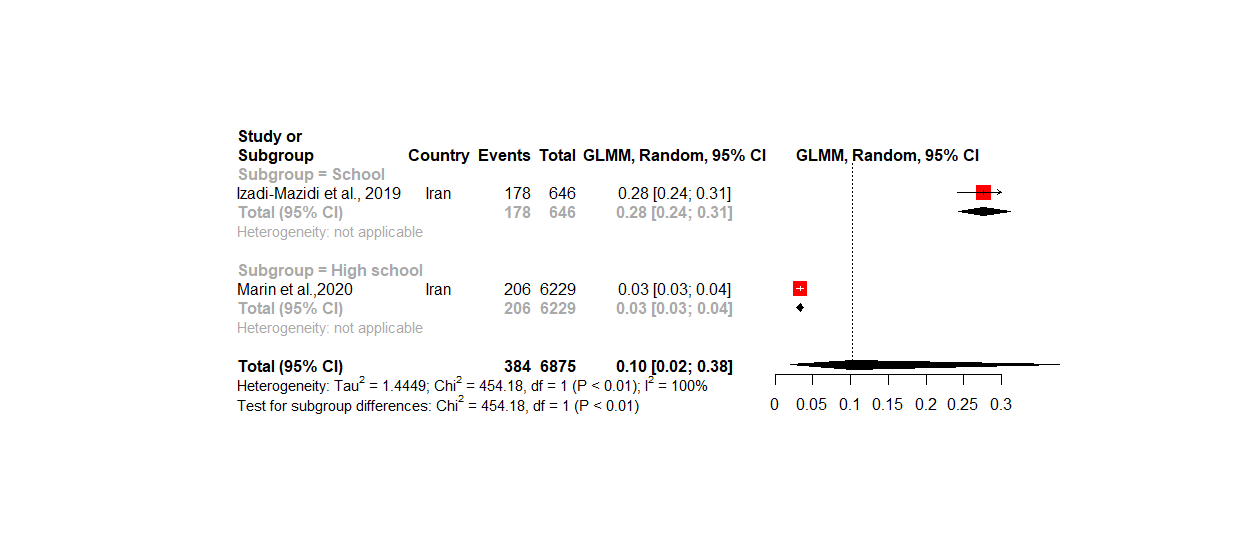


Supplementary file 3B: Pooled estimate for the prevalence of NSSI in past year
